# Feasibility of IMU-Based Wearable Sonification: Toward Personalized, Real-Time Gait Monitoring and Rehabilitation

**DOI:** 10.3390/bios15100698

**Published:** 2025-10-15

**Authors:** Toh Yen Pang, Chi-Tsun Cheng, Frank Feltham, Azizur Rahman, Luke McCarney, Carolina Quintero Rodriguez

**Affiliations:** 1 Biomedical Engineering Department, School of Engineering, STEM College, RMIT University, Melbourne, VIC 3000, Australia; 2 Mechanical, Manufacturing and Mechatronic Engineering Department, School of Engineering, STEM College, RMIT University, Melbourne, VIC 3000, Australia; ben.cheng@rmit.edu.au; 3 Industrial Design, School of Design, College of Design and Social Context, RMIT University, Melbourne, VIC 3000, Australia; frank.feltham@rmit.edu.au; 4 Occupational Health and Safety/Ergonomics, School of Property, Construction and Project Management, Design and Social Context, RMIT University, Melbourne, VIC 3000, Australia; azizur.rahman@rmit.edu.au; 5 School of Health and Biomedical Science, Health Sciences Osteopathy RMIT University, Bundoora West Campus, Bundoora, VIC 3083, Australia; luke.mccarney@rmit.edu.au; 6 Enterprise, School of Fashion and Textiles, RMIT University, Brunswick Campus, Brunswick, VIC 3056, Australia; carolina.quinterorodriguez@rmit.edu.au

**Keywords:** biofeedback, user-centered design, biomechanics, neurorehabilitation, digital biomarkers

## Abstract

Wearable auditory feedback systems have demonstrated potential to support gait rehabilitation, yet user experience and engagement remain underexplored. This feasibility study investigated the usability and perceptions of an IMU-based (WT901BLECL, WitMotion) sonification system designed to deliver real-time gait feedback. Twenty healthy participants walked on a treadmill at two speeds under three conditions: no feedback, discrete bass tones, and continuous whoosh tones. The proposed system, with an IMU sensor embedded in a flexible garment, combined real-time gait analysis with auditory cues. Participants reported high levels of comfort, with most (90%) indicating that they had a positive overall experience. Discrete bass tones enhanced awareness of specific gait phases, particularly heel strike and initial contact, whereas continuous whoosh sounds extended awareness to the trunk and hips but were occasionally perceived as distracting. Motivation effects were mixed, and no significant correlations were found between subjective ratings and biomechanical measures, reflecting individual variability in auditory cue interpretation. These results emphasized the role of sound modality in influencing gait perception and highlighted the importance of user-centered design in wearable rehabilitation technologies. The study provides foundational evidence for refining personalized auditory feedback systems and supports future investigations with clinical populations, such as stroke survivors and individuals with Parkinson’s Disease.

## 1. Introduction

As populations age and chronic health conditions become more prevalent, the demand for innovative rehabilitation technologies is growing rapidly. For individuals with mobility impairments caused by aging or long-term illnesses, such as stroke or neurological disorders, the ability to maintain mobility is often diminished, significantly impacting independence and quality of life [1]. Traditional rehabilitation programs, while effective, often rely on intermittent therapist-delivered feedback during scheduled sessions. This arrangement introduces challenges relating to time, cost and accessibility [2], leading to reduced patient compliance, motivation and slower progress, ultimately resulting in extended or ineffective treatment cycles [3].

Wearable feedback systems have therefore emerged as practical tools for biomechanical assessment and movement enhancement, particularly in gait analysis and rehabilitation [1,4,5,6,7,8]. These devices typically focus on detecting distinct gait events, such as heel strikes or freezing of gait [7,9,10], and some extend to cueing interventions through either audio or visual prompts [11,12,13]. However, most existing systems are designed to react to abnormal gait patterns rather than proactively enhancing gait awareness and stability [9,14]. They often overlook the critical role of user perception, comfort and engagement in determining whether such systems will be adopted in everyday rehabilitation [15,16]. Furthermore, existing technologies are designed primarily for monitoring patients, rehabilitation or fall prevention, rather than promoting self-awareness or preventive gait enhancement [17,18]. Few systems offer immediate feedback, leveraging a human’s capacity to interpret auditory data on the efforts and movement of the limbs in space, intended to improve technique, balance, or overall posture for the prevention or optimal performance of walking [19].

Auditory feedback (sonification) has recently gained attention as a method to translate gait kinematics into sound cues that guide users’ movement [5,13,20,21,22,23]. Unlike musical cueing methods, which rely on rhythm or pre-recorded tracks and may be cognitively demanding [24,25,26], sonification enables real-time, intuitive auditory signals tailored to an individual’s movement. By modulating sound features such as pitch, timbre and amplitude, auditory feedback can improve step timing and motor balance, with clinical studies reporting benefits for patients with Parkinson’s Disease and stroke [27,28,29]. Unlike visual feedback, which can be distracting or cognitively demanding, auditory biofeedback operates in parallel with movement, facilitating proprioceptive awareness and continuous monitoring during activity. For example, Pauletto et al. [30] emphasized the importance of sound design in rehabilitation, noting that continuous and audio feedback can enhance user engagement and perception. Braun Janzen et al. [25] found that rhythmic auditory cues are able to facilitate motor learning, improve step timing and coordination in individuals with gait impairments. Varlet et al. [27] reported that low-pitched auditory rhythms induced stronger spontaneous entrainment of participant movements compared to high-pitched rhythms. These cues also supported movement stabilization. Shin et al. [28] also reported that rhythmic auditory stimulation gait training enhances kinematic and temporospatial gait parameters, including hip adduction, knee flexion and ankle plantar flexion. Furthermore, Rhythmic Auditory Cueing (RAC) has been explored for its effectiveness in facilitating motor rehabilitation for patients with stroke and neurological conditions [28,31,32,33,34]. However, the results for the effectiveness of RAC and its intervention efficacy are mixed, and the existing sonification systems typically use generic cues (e.g., fixed metronome beats or standardized thresholds) rather than tailoring signals to the individual’s gait cycle or movement style [35,36,37]. This lack of personalization in the auditory cue design limits the system’s effectiveness and may reduce user engagement, as generic sounds may not resonate with or optimally guide the user [20,38].

A rapidly emerging direction in personalized sonification, which adapts the timing, type and acoustic properties of cues to an individual’s sensory, motor and motivational profile [5,20,38,39]. Recent studies demonstrate that tailoring sound cues to user-specific gait parameters or perceptual preferences, such as matching walking rhythm with sound beats or adjusting pitch, can improve motor learning, enhance stability and sustain long-term engagement compared to generic or non-adaptive feedback [5,20,38,39]. Personalized cueing thus represents a shift from uniform auditory interventions to adaptive systems that are responsive to users’ physiological and psychological needs.

However, a challenge lies in the complexity of the auditory feedback itself. Systems that embed cues within music may limit accessibility, as users unfamiliar with musical structures may struggle to connect their movements with the changing sounds, and consequently reduce user engagement and the overall efficacy of the intervention [25,26]. This highlights a need for simpler, real-time, and more intuitive sonification approaches that support user understanding and encourage active gait correction. Most studies emphasize quantitative gait improvements but rarely capture the subjective experience on how users perceive and internalize these cues and how this influences their movement consistency beyond quantifiable metrics, potentially tapping into “felt experience”, which are critical for long-term adoption as proposed by Thorn and Willcox [40]. Furthermore, gaps remain in understanding how users perceive such feedback, what kinds of auditory cues are most engaging [20], and how wearable systems can strike a balance between technical accuracy, comfort and usability [41].

To understand this new development, it is important to compare RAC with sonification. RAC uses a regular beat or timed pulses to help a person synchronize their movement, such as marching to a metronome. This technique is effective in driving synchronization through entrainment. However, sonification offers a different approach. It translates specific movement data (parameters) into either discrete or continuous, real-time signals, providing dynamic feedback. This approach offers greater support for complex or variable movements that require constant adjustment. The comparison in Table 1 highlights the differences in feedback type, mapping strategies, adaptivity and engagement potential.

Despite promising findings, several gaps remain in the literature. To address these gaps, we developed and evaluated a novel IMU-based wearable sonification system that generates and delivers personalized real-time auditory feedback during gait for each user. The system provided two forms of auditory feedback: (i) The continuous auditory feedback consisted of tones that varied in “brightness” or pitch according to the participant’s trunk rotation relative to a pre-defined reference region. As the participant moved further from this reference region, the tones brightened, signaling increased displacement. In contrast, the tones dulled when movement remained close to the reference, providing an intuitive cue of reduced deviation. This design enabled participants to continuously monitor their posture in real time through gradual tonal changes. (ii) The discrete bass tone was incorporated as an alert mechanism. This sound was triggered whenever trunk rotation exceeded the individualized threshold established from baseline walking, clearly indicating that the user had moved outside their reference region. The detailed design and rationale of this personalized auditory feedback system have been reported in our previous study [5].

The ultimate goal of this system is to support clinical populations such as stroke survivors and individuals with Parkinson’s Disease by enhancing their movement awareness and facilitating rehabilitation outside of traditional clinical settings. However, as a necessary first step in the development of rehabilitation devices [52], this study tested the system in a controlled trial with healthy participants. This feasibility study aimed to:

1. Evaluate the comfort, usability and acceptability of the wearable system.
2. Explore whether auditory feedback motivates users to adjust gait kinematics (e.g., trunk sway, symmetry, or maintain stability).
3. Establish a baseline understanding of perceptual responses to real-time sonification before transitioning to patient populations.

By integrating user-centered design with personalized, real-time auditory cues, this study demonstrates how simplified, real-time auditory feedback can increase movement awareness even in healthy individuals, thereby validating the feasibility and user acceptance of the approach. These findings provide the foundation for clinical application, where such a system could deliver continuous, personalized and motivating feedback to patients who need to retrain their gait.

## 2. Materials and Methods

The project commenced with designing and developing customized wearable sensors capable of tracking limb movements and collecting real-time gait data.

### 2.1. Participants

Twenty healthy participants aged between 21 and 60 years (Table 2) were recruited to participate in the study. They were recruited from the university community using flyers posted around campus, which outlined the purpose and procedure of the study. Interested individuals were invited to contact the research team and schedule a laboratory session in the Biomechanics Laboratory. Eligibility was confirmed through self-report at enrolment, based on predefined inclusion (age 18–65, good health, adequate hearing and able to walk on a treadmill) and exclusion (any musculoskeletal, neurological, cardiovascular, or hearing conditions that affect safe participation) criteria.

There were twelve males and eight females who self-reported with no existing injuries or had undergone major surgeries to the lower limbs in the last three months that prevented them from performing a regular walk on a treadmill.

The sample size (N = 20) was determined in accordance with the aims of this study, which included feasibility and usability evaluation. According to Teresi et al. [53] feasibility studies should prioritize estimating outcomes such as protocol completion and acceptability, rather than hypothesis testing. Furthermore, the sample size was sufficient to achieve thematic saturation based on 9–17 participant-reported experiences [54].

This study was conducted in accordance with ethical guidelines and was approved by the RMIT College Human Ethics Advisory Network. All participants volunteered for this research and were provided with a detailed information sheet clearly outlining the required activities and procedures involved. Informed written consent was obtained from each participant prior to their involvement.

### 2.2. The Sensor-Based Wearable System

The wearable consisted of a single inertial measurement unit (IMU) (WT901BLECL, WitMotion, Shenzhen, China) sensor embedded into a lightweight, flexible garment. The IMU integrated a 9-axis accelerometer, gyroscope and magnetometer, with data transmitted via Bluetooth 5.0. The data output was configurable at sampling frequencies of up to 200 Hz (default: 10 Hz) with an attitude measurement accuracy of 0.2°. The accelerometer had a measurement range of ±16 g with an RMS noise of 0.75–1 mg, and the gyroscope operated at ±2000°/s with an RMS noise of 0.028–0.07°/s. The magnetometer had a measuring range of ±2 Gauss with a resolution of 0.0667 mGauss/LSB.

The garment was made from a commercially available fabric (95% Polyester and 5% Spandex) secured with adjustable Velcro fastening and attached around the waist like a large soft belt or band. The sensor was positioned around the L4 region of the participants’ lower back to track limb movements during walking tasks. Such a design choice ensured the wearable system did not interfere with the natural range of motion during exercises. The IMU sensor was configured with a sampling rate of 100 Hz and was calibrated to detect movement. Raw acceleration and angular velocity signals were smoothed with a 4th-order Butterworth low-pass filter at 6 Hz to retain gait dynamics, such as heel strike and toe-off, while attenuating high-frequency noise [55]. Data from the sensor were transmitted to a computer for processing.

We developed algorithms to analyze gait data in real-time and generate immediate auditory feedback. Our approach was established via previous studies [5,56]. These algorithms were designed to detect deviations from baseline movement patterns established through pilot testing.

### 2.3. Personalized Auditory Feedback for Gait Data

The personalized auditory feedback system utilized gait data from the IMU sensor to generate real-time auditory cues tailored to each user’s walking movement patterns. The sensor captured sagittal plane rotations, which were then analyzed in MATLAB (R2022b, MathWorks, Natick, MA, USA) to establish individualized baseline thresholds for detecting significant deviations during walking or balancing.

Heel strikes were identified using a turning-point detection method based on time-series rotation angles. The algorithm evaluated local peaks by combining [5]:

$x=\left(a\left[n-1\right]-a\left[n-2\right]\right)\times \left(a\left[n-2\right]-a\left[n-3\right]\right)$, to determine a turning point;

$y=\left(a\left[n-1\right]-a\left[n-2\right]\right)$, to determine the trend direction.

A positive *xy* value indicated a heel-strike event. To reduce false positives, a threshold window was computed from each participant’s no-audio baseline trial, ensuring individualized thresholds. A baseline gait profile was established during the initial no-audio walking condition, from which personalized thresholds were computed automatically in MATLAB for each participant.

**Figure 1:**
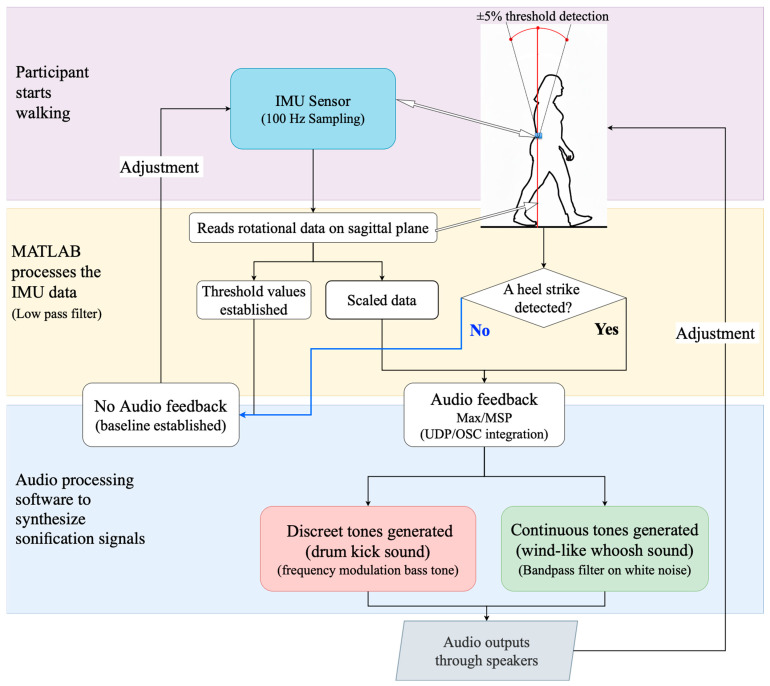
Flow diagram for data collection with IMU sensor and sonification generation for auditory feedback.

These data were transmitted to the Max/MSP programming environment, version 8 (Cycling ’74, Covina, CA, USA). As the participant walked, sound was generated. When a deviation was detected, the gait information was translated to initiate two distinct sound events (Figure 1).

The sonification system was designed to provide participants with both continuous and discrete auditory feedback linked to torso rotation in the sagittal plane. Following calibration, a baseline (reference datum plane) was derived from the participant walking normally without auditory feedback, and individualized thresholds were set at ±5% deviation from this datum plane [56].

The first sound event consisted of a discrete bass tone with a sharp attack (we referred to this as the “bass sound” for clarity). This bass tone was similar to a kick drum in electronic music. The bass tone was implemented as a sine wave with a fundamental frequency of 440 Hz and a duration of 800 ms [56]. This tone alerted the user to specific events, such as heel strikes or significant loss of balance, providing clear corrective cues. Simultaneously, a second sound event in the form of a wind-like continuous tone (i.e., we referred to this as the “whoosh sound”) was created by adjusting the pitch and brightness relative to the participant’s movement within or outside a reference plane. Rotation values within the threshold region were mapped to the center frequency of a band-pass filter applied to a databank of tuned oscillators in Max/MSP. As the person moved further away from the reference plane, the filter frequency shifted upward, brightening the tone. As rotation decreased, the tone became duller. This tone offers auditory feedback that helps the user make minor adjustments to maintain stability through the phases of the walk cycle. This real-time auditory feedback helped participants understand their balance state and make necessary adjustments during movement.

### 2.4. Data Processing and Sonification

The data processing and sonification workflow (Figure 1) began with capturing angular rotation on the sagittal plane, focusing specifically on forward and backward tilt motions during walking.

Raw sensor data were collected and processed in real-time, including filtering and extracting key gait features (e.g., detecting heel-strike events). The processed data then drove a real-time sonification algorithm designed in Max/MSP, converting gait metrics into auditory signals (bass or whoosh sounds). The IMU, MATLAB and Max/MSP were connected locally via TCP/IP and Open Sound Communication protocols. This auditory feedback was immediately provided to participants via speakers to enhance awareness and motivation during walking.

### 2.5. Experimental Protocol

**Figure 2:**
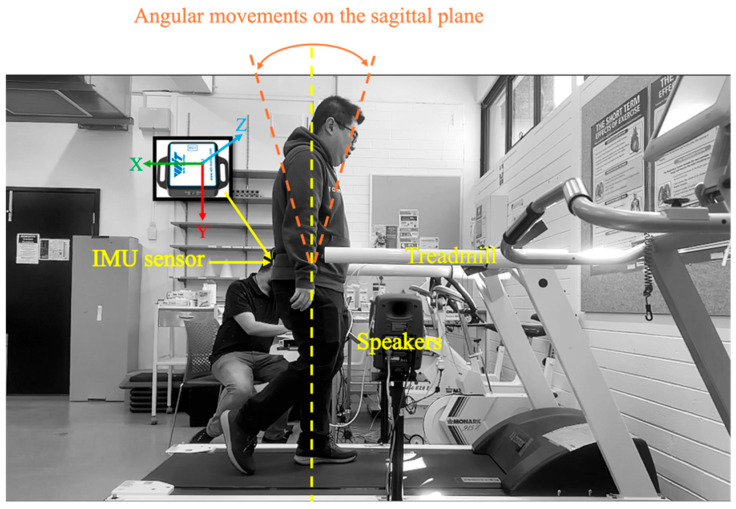
Experimental setup for gait analysis with sonification feedback.

The experiment was conducted in the Biomechanics Laboratory of RMIT University (Figure 2). Participants were invited to walk on the treadmill, to establish a stable and natural gait before data collection. Each participant was first allowed one to two minutes of walking on the treadmill at a self-selected, comfortable pace. This arrangement allowed participants to adjust to their movement patterns and establish a consistent walking rhythm. Once they were familiar with the treadmill environment, data collection commenced.

The sonification feedback was generated based on the real-time analysis of participants’ gait data and delivered through two external speakers (Genelec 80200D Studio Monitor by Genelec Oy, Iisalmi, Finland), mounted on adjustable floor stands. The speakers were positioned on the left and right sides of the treadmill at waist height. These speakers provided a wide frequency response (56 Hz–25 kHz, −6 dB) and high output with minimal distortion. As the study was conducted in a controlled laboratory environment, this ensured that participants consistently received the sonification signals under each feedback condition without any interference. A video was placed on the side to capture the movement footage for further analysis.

In the no-audio condition, baseline gait data were collected to establish individual walking patterns. These baseline measurements were essential for calibrating the system and identifying unique threshold values for each participant using MATLAB software, which were later used to personalize the sonification feedback.

### 2.6. Data Collection and the Questionnaires

During the experiment, participants were instructed to complete six trials, consisting of two fixed treadmill speeds—2 km/h and 3.9 km/h. The lower speed of 2 km/h approximates a slow, comfortable pace, comparable to the range of gait velocity observed among elderly adults. The higher speed of 3.9 km/h aligns with average normal walking speeds reported for healthy elderly populations [57]. Participant also walked with three different auditory feedback configurations: (1) ‘no-audio’ feedback that served as a baseline measure, (2) discrete audio tones (i.e., base sound), and (3) continuous audio tones (i.e., whoosh sound).

At the slower speed (2 km/h), participants first walked without auditory feedback, followed by the discrete tone (a bass sound), and finally the continuous tone (a whoosh sound). The same sequence was then repeated at the faster speed (3.9 km/h). This fixed ordering was adopted for two reasons: (1) it aligned with the questionnaire design, enabling participants to recall and compare their experiences in different trial conditions accurately, and (2) it reduced potential confusion by providing a clear progression through the unfamiliar auditory feedback modes.

After completing the walking tasks, participants completed a questionnaire to evaluate their subjective experiences with the auditory feedback system. The questionnaire consisted of quantitative and qualitative components, using both 5-point Likert scale specific to assessing comfort, motivation and awareness [9,58,59]. Specifically, participants rated their experiences addressing the following dimensions:

4. Comfort: How do you feel about the wearable device? How do you rate the comfort level?
5. Motivation: Did the auditory feedback motivate you to walk?
6. Awareness: Did the auditory feedback make you more aware of your movement in real time?

In addition to the Likert scale items, participants were asked open-ended questions to capture more detailed feedback on their overall experience, including suggestions for improvement and any challenges they encountered.

While subjective feedback from the questionnaire was not a direct measure of kinematic or kinetic variables, it helped us to understand the context and user-centered validation, such as their experience and acceptance of a wearable system that intended to improve mobility or to modify their movement patterns (kinematics outcomes) [41].

### 2.7. Data Analysis

IBM SPSS Statistics for Windows, version 29 (IBM Corp., Armonk, NY, USA), was used to perform the data analyses, including basic descriptive statistics and determining the significant relationships between variables. Descriptive statistics (mean, standard deviation (SD), and range) were computed to summarize the participants’ ratings for auditory stimuli in various subjective ratings such as comfort, awareness and motivation.

The normality of the data was checked as a preliminary requirement to determine whether parametric or non-parametric tests should be applied. A paired samples *t*-test was considered for normally distributed data. Otherwise, the Wilcoxon signed-rank test was selected. Correlation analyses were performed between subjective ratings and the biomechanical variable measured (angle of rotation on the sagittal plane). Pearson’s correlation coefficient (*r*) was applied if data met the normality assumption; otherwise, Spearman’s rank-order correlation (*r*) was used [60]. A *p*-value of 0.05 was used when comparing variables.

**Figure A1:**
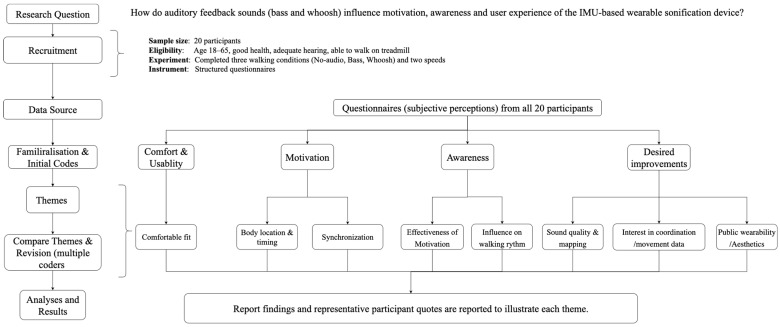
Flow diagram of the qualitative analysis process, showing progression from the research question and data source through initial codes to higher-level themes and results on motivation, awareness and comfort about the IMU-based wearable sonification prototype.

The qualitative responses were analyzed to identify common themes regarding the participants’ subjective experiences regarding auditory stimuli (bass and whoosh sounds) and the wearable device’s comfort, functionality, and its impact on gait awareness and motivation. Following the approach by Braun and Clarke [61], thematic analysis was employed to systematically identify, analyze, and report themes within the qualitative dataset (refer to Figure A1). Two coders independently reviewed and coded the transcripts to capture participant perspectives. Coding frameworks were discussed iteratively, and discrepancies were resolved through consensus meetings until full agreement was reached.

## 3. Results

### 3.1. Comfort

**Figure 3:**
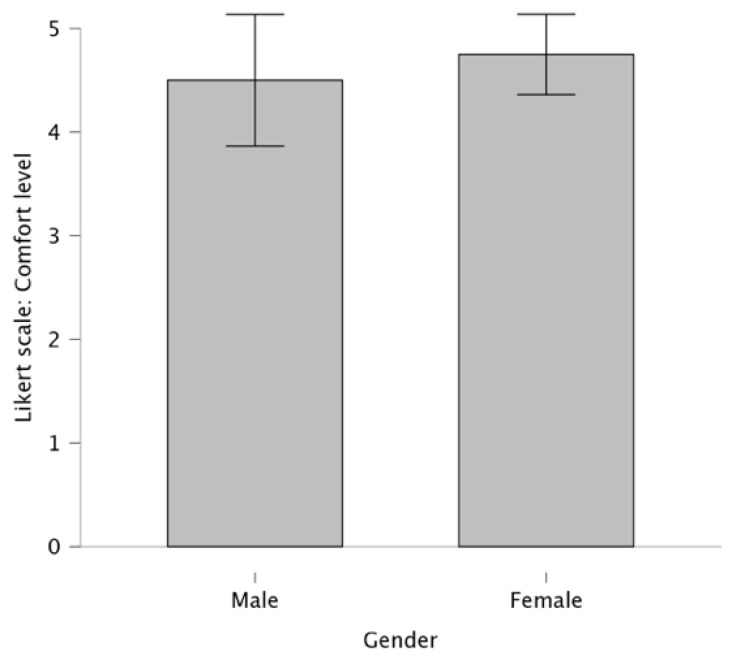
Comparison of comfort ratings between female and male participants (the bars represent the mean values; the error bars represent the corresponding SD).

The comfort level of the wearable device was rated by 20 participants on a scale from 1 to 5 (Figure 3). The majority (90%) of participants gave an average score of 4.6 on the 5-point Likert scale (N = 20, M = 4.6, SD = 0.8), highlighting a consensus that the wearable device was comfortable. Only 10% of participants rated the comfort as 2 or 3, suggesting they found the devices less comfortable. No significant differences were found between female and male participants (*p* > 0.05).

The majority of participants, about 90%, reported a positive experience with the wearable device in terms of comfort, with no significant interference during the walking tests. Many noted that the device was lightweight, with several participants mentioning they forgot they were even wearing it. The ability to adjust the hip-band or waist strap to achieve the right level of tightness was appreciated, as it contributed to the overall comfort.

### 3.2. Motivation

**Figure 4:**
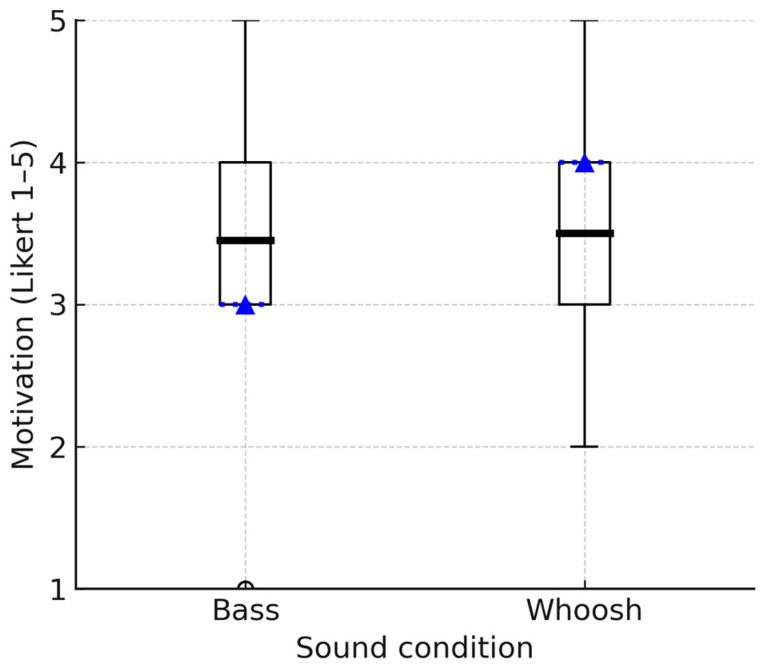
Boxplots comparing the distributions of motivation ratings for bass and whoosh sounds. The box indicates the interquartile range (IQR); the dotted line represents the median, the bold solid line represents the mean, and the whiskers represent 1.5 × IQR.

The effectiveness of two auditory stimuli—the bass and whoosh sounds—were analyzed in relation to their ability to motivate participants to engage in walking. The mean motivation rating for the bass sound was 3.45 (SD = 0.9), suggesting a moderate overall effectiveness in motivating participants to walk. The mean motivation rating for the whoosh sound was slightly higher at 3.50 (SD = 1.0) (Figure 4).

A Wilcoxon signed-rank test was used to assess whether there was a significant difference between the motivational ratings of the two auditory stimuli. The differences between the two groups are minimal (Z = −0.120), and there is no statistically significant difference between the two compared groups. In other words, the motivation ratings for the two auditory stimuli (bass and whoosh sounds) are not significantly different (*p* > 0.05).

Based on the participant feedback for both the bass and whoosh sounds in terms of their motivational impact on walking, two common themes emerged for each sound:

- Theme 1: effectiveness of motivation. The participants’ responses regarding the motivational efficacy of the bass and whoosh sounds indicated a mixed impact, with a considerable proportion reporting positive and negative effects for each sound. For example, half of the participants (50%) stated that the bass sound did not enhance their motivation and, in fact, negatively affected them. Participants described the sound as confusing, jarring, or not in sync with their walking rhythm. An excerpt from a participant: “*I found the bass sound jarring, was not conducive to the rhythm of walking*” (P3). Similarly, 50% of participants reported that the whoosh sound neither motivated nor negatively impacted them. They described the sound as confusing, disorienting, or distracting. One participant stated: “*The whoosh sound did not have any particular effect on my motivation*” (P1). 45% (9 participants) found the bass sound to have a positive motivational impact. These participants noted that the sound encouraged alertness, increased movement speed, or provided a sense of preparedness. “*It made me prepare to be alert during walking. Really sound made me motivated to move forward*” (P14). Similarly, 40% described the whoosh sound as having a positive motivational effect, primarily by increasing their pace or improving movement rhythm. One participant stated: “*The woosh sound made me feel I wanted to walk faster to increase motivation to walk faster*” (P17).
- Theme 2: the influence on walking rhythm. For example, 40% reported that the bass sound increased their awareness of the timing of foot strike and gait cycle. However, the timing did not align well with their natural rhythm. One participant stated: “*It makes me aware of my gait cycle and foot strike—didn’t change my motivation to participate in walking*” (P7). Another 45% noted that the whoosh sound disrupted their rhythm or made it difficult for them to align with the sound. Another participant stated: “*I felt like a distraction than walking. I couldn’t tie my walking to the sound as it felt random. I think I lost a bit of concentration*” (P8).

### 3.3. Awareness

**Figure 5:**
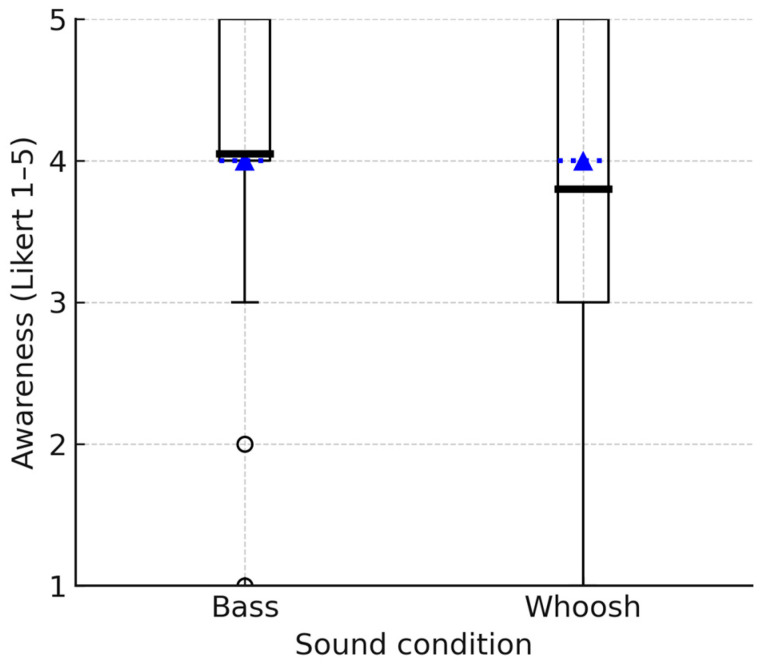
Boxplots showing the group distribution of awareness ratings for bass and whoosh sounds. The box indicates the IQR; the dotted line represents the median, the bold solid line represents the mean, and the whiskers represent 1.5 × IQR.

Figure 5 illustrates the distribution of ratings given by participants for the bass and whoosh sounds, assessing their effectiveness in making participants more aware of their movement. The mean awareness rating for the bass sound was 4.05 (SD = 1.1). This result indicates that, on average, participants perceived the bass sound to be effective in making them aware of their movements, leaning towards the higher end of the awareness scale. The mean awareness rating for the whoosh sound was 3.80 (SD = 1.2), which is slightly lower than the mean for the bass sound. However, the Wilcoxon signed-rank test results indicate that the two groups have similar distributions and no statistically significant difference between the ratings of movement awareness for the bass and whoosh sounds (Z = 0.896, *p* > 0.05).

Based on the participants’ responses regarding awareness when exposed to bass and whoosh sounds, two common themes emerged for both auditory stimuli: awareness of the body’s location and timing and the impact of sound synchronization with movement. For the bass sound, based on the responses provided, the analysis of participants’ awareness during the use of auditory stimuli for movement feedback revealed two common themes:

- Theme 1: awareness of the body’s location and timing. For the bass sound, 80% reported that their awareness was predominantly at the beginning of the footstep cycle or during the heel strike phase. 70% specifically reported awareness in their feet or legs. “*In some instances, I almost followed the beat’s rhythm. The awareness came in the lower leg*” (P16). For the whoosh sound, 70% reported an increase in awareness either at the beginning of their gait cycle or during the swing phase of their legs, and 35% reported awareness in areas such as the hip or lumbar spine. “*Hip—during swing forward. The sound is long enough to incorporate the entire movement”* (P3).
- Theme 2: impact of movement synchronization with sound. For the bass sound, 50% reported that the bass sound was either effective or partially effective in aligning with their gait rhythm, which led to increased awareness. Participants noted that the sound made them aware of their gait cycle or encouraged them to focus on foot strike. “*The bass feels more effective, especially when it is in sync with the gait which is mostly the state of the gait cycle. The soundtrack feels more effective on leg level*” (P10). For the whoosh sound, 45% found it helpful to their gait rhythm, making them more aware of their movements. “*In the beginning, the woosh sound made me expect wind and a need to correct my gait to adjust to the wind*” (P2).

### 3.4. Impact of Audio Feedback on Gait

**Figure 6:**
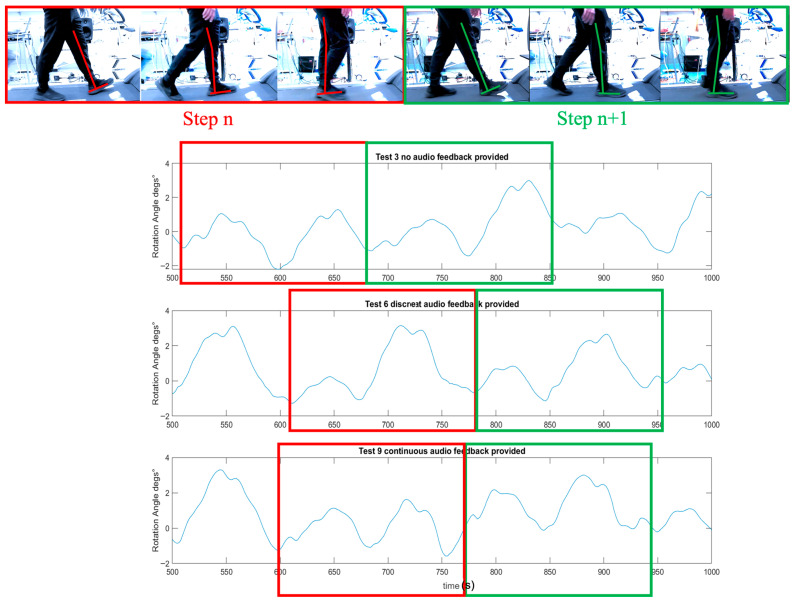
The representative’s rotation angle (°) on the sagittal plane for a participant when walking under three auditory conditions (no audio, discrete bass sound and continuous whoosh sound) on a treadmill. The graphic overlay (red and green line) represents the reference leg and corresponds the angle of rotation measured on the sagittal plane during gait.

Figure 6 illustrates the rotation angle of the representatives on the sagittal plane, measured by an IMU sensor, under three auditory conditions (no audio, discrete bass sound, and continuous whoosh sound). Both auditory feedback conditions demonstrated a more rhythmic and consistent rotation profile than the no-audio condition. However, a closer examination revealed a distinction between the two auditory conditions. Specifically, the discrete bass sound condition exhibited a rotation angle pattern that was more symmetrical in two consecutive steps. This symmetry suggested a more balanced and predictable gait pattern. The regularity and mirror-like quality of the rotation angle peaks and troughs in the bass sound condition implied that participants were likely more aware of their gait cycle, potentially due to the rhythmic cues provided by the discrete bass. In contrast, while the continuous whoosh sound also improved rhythmicity, it did not produce the same level of step-to-step symmetry, indicating a potentially different impact on gait control and awareness.

In the Shapiro-Wilk test for normality, the rotation angle on the sagittal plane follows a normal distribution. Pearson correlation analyses were conducted to investigate the relationships between the mean angles of rotation and subjective measures (motivation and awareness) under discrete (bass) and continuous (whoosh) auditory feedback and two different walking speed conditions. The results are summarized in Table A1.

Although results indicated that subjective ratings of motivation and awareness were not significantly related to measured changes in the angle of rotation in the sagittal plane during walking tasks (Table A2), this finding highlighted the complexity of user perceptions and their relationship with biomechanical outcomes.

### 3.5. Users’ Overall Feedback and Areas for Improvement

While the wearable device was generally well-received by participants, it also offered insightful suggestions on further improving their user experience, emphasizing elements such as customizability, functionality and real-world applicability.

Several (25%) participants noted that the whoosh or bass sound could be more effective if it matched their steps, suggesting a more intuitive, interactive experience. Participant excerpt: “*Could the whoosh align with my steps, that might fit better with awareness of my gait.*” (P4).

Additionally, 15% of the participants showed interest in gaining more insight into their coordination and movement data while using the device. They sought a deeper understanding of the purpose behind movement awareness and how to derive benefits from it. Participant excerpt: “*It would be great if I could understand when I am slightly uncoordinated out of interest*.” (P8).

Some participants (20%) expressed concerns about using the belt in public due to its visibility, suggesting a more aesthetically pleasing design. Participant excerpt: “…*Wearing the belt would make me feel conscious when in public.*” (P1).

## 4. Discussion

This study extended the previous work reported previously [5] by incorporating subjective user experiences, such as comfort, motivation and awareness, into the evaluation of a wearable auditory feedback system. Individual variability in perceptions and responses to auditory feedback may affect the biomechanical adjustments captured by wearable technology; understanding these subjective dimensions is essential for future clinical adoption. The current study explored the impact of two distinct auditory modalities, i.e., bass (discrete) and wind-like whoosh (continuous) sounds, on participants’ movement awareness, motivation and biomechanical parameters, such as rotation angle during gait.

The chosen auditory stimuli were selected based on their rhythmic characteristics: (1) the bass sound represented a low-frequency, rhythm-based stimulus previously linked to motor synchronization, while (2) the whoosh sound offered a continuous, non-rhythmic stimulus for broad movement awareness. Results showed that the bass sound was particularly effective in creating awareness at specific gait events, such as heel strike and initial contact. In contrast, the whoosh sound appeared to extend awareness beyond the lower limbs to include the hips and spine. Despite similar influences on motivation, participants perceived inconsistencies in the rhythm of the bass sound, which occasionally reduced its motivating effect, while the whoosh sound was distracting or destabilizing for some individuals. These findings demonstrated how the discrete and continuous sounds affected participants’ perception of movement and their level of engagement with the sonification system.

### 4.1. Healthy Participants and Feasibility

The use of healthy individuals presented interpretive challenges, since they normally do not experience gait deficits or discomfort that would necessitate adjustment. As expected, the study did not reveal large biomechanical improvements. However, healthy participants provided a safe and controlled setting to test the system’s technical accuracy and validate that the sonification process works correctly. This study also captured subjective experiences to understand how users perceived the auditory feedback without the added complexity of clinical conditions. Importantly, even in participants without gait issues, the sounds caused slight adjustments in their walking and made them more aware of their movements, demonstrating that the system can influence a person’s walking in real-time. This feasibility phase was a necessary step before conducting clinical trials with stroke survivors, older adults and individuals with movement disorders. The study provided important insights that allowed us to refine the intervention, as suggested previously [52], before applying it to these patient groups, which will have a greater scope and potential for rehabilitation benefits.

### 4.2. Evaluation Criteria: Motivation and Awareness

We acknowledged that healthy participants are expected to become more attentive when exposed to external stimuli. In this feasibility study, however, measures of awareness and motivation were selected as indicators of usability, engagement and acceptability. These factors were essential for ensuring long-term adherence to rehabilitation technologies. Participants reported that rhythmic bass sounds helped them focus on specific gait events (e.g., initial foot contact), while continuous ‘whoosh’ sounds promoted a sense of whole-body awareness during walking. Awareness ratings were generally high (median = 4 for both sound types), supporting the device’s usability and perceived ability to draw attention to movement.

However, the statistical analyses of the angle of rotation on the sagittal plane at 2 km/h and 3.9 km/h revealed no significant differences between the auditory and no-audio conditions (all *p* > 0.05, and with small effect sizes). These findings indicate that while auditory feedback enhanced subjective awareness, it did not objectively improve gait rhythmicity or symmetry in this healthy cohort.

Importantly, 40% of participants also reported that certain sound types occasionally caused a sense of imbalance, emphasizing inter-individual variability in perceptual responses. This outcome highlights the need for developing personalized auditory feedback systems that can adapt to user preferences and movement characteristics, a direction our study helps to inform by demonstrating both the potential and the limitations of generic sonification approaches.

Participant feedback also indicated that the sound occasionally felt “confusing” or misaligned with their rhythm, which may indicate (a) a potential lag in the sensing-to-sound pipeline and/or (b) missed or sub-optimally mapped rotation on the sagittal plane with a single IMU. Although audio playback was continuous during experiments, we did not rule out timing offsets. Future studies should quantify latency in experimental trials and verify the capture and mapping of sagittal-plane rotation to ensure temporal alignment with gait events.

### 4.3. Implication for Rehabilitation

This study integrated a lightweight wearable sensor, a flexible garment-based IMU system, and a user-centered sonification design. The IMU-based system was able to capture gait features, such as step timing and trunk rotation. These are increasingly recognized as digital biomarkers that provide objective, quantitative indicators of mobility. Such biomarkers are important for tracking disorder progression in neurological conditions such as stroke and Parkinson’s Disease [8].

Thus, the system served a dual function: (1) intervention, by providing immediate auditory feedback to promote motor learning and awareness; and (2) monitoring, by logging gait parameters that could inform clinical decisions and track rehabilitation progress.

Our findings suggested that the bass sound could be highly effective for refining specific gait phases, such as heel strike and initial contact. They may be particularly beneficial for individuals seeking to improve balance or accuracy during these critical movements. Meanwhile, non-rhythmic whoosh sounds may promote overall body awareness and assist with posture and balance. These insights could inform the design of tailored auditory feedback systems that tailor to individual rehabilitation needs, improving engagement and motor learning outcomes. For example, lower-frequency bass cues may support targeted retraining of heel strike symmetry in stroke survivors, while continuous cues could assist in improving postural awareness in balance-impaired patients.

In addition to focusing on the algorithmic accuracy, the system was evaluated for its ability to deliver personalized sound feedback for gait and movement. Our system demonstrated real-time monitoring of gait parameters and translated these into distinct auditory modalities designed to enhance user engagement.

The individualized interpretation of auditory stimuli appears to play a crucial role in their effectiveness. Participants expressed a desire for greater customizability of sound feedback, with many suggesting that aligning the auditory feedback more closely to their gait would enhance its effectiveness. The finding highlighted the importance of aligning auditory cues with user expectations and natural movement patterns to ensure efficacy.

This integration of signal processing, real-time biomechanics, and user-perception evaluation represents a significant advance towards developing practical and portable rehabilitation technologies.

### 4.4. Limitations and Future Directions

Although the present study provides initial insights into the feasibility of wearable auditory feedback, the statistical interpretation is limited by the small and homogenous sample size (N = 20), which predominantly comprises young, healthy adults, and the short-term nature of the intervention. This composition may limit the generalizability of our findings to broader populations, particularly older adults or those with mobility impairments. With such a small sample, statistical power to detect subtle differences between auditory conditions is limited, and effect size estimates should be interpreted with caution due to wide confidence intervals [53].

Comfort ratings in this study reflect subjective participant perceptions about the IMU-based wearable prototype; however, they did not capture objective aspects of comfort. Future work should focus on integrating objective measures, such as pressure distribution, skin temperature and any movement restriction, into evaluations of our device as it is refined.

Additionally, we did not control for participants’ prior exposure to different auditory stimuli, which may have introduced variability in the responses observed. Future research should involve more diverse participant samples, and a deeper investigation into the role of prior auditory experiences could shed light on how these factors influence responses to auditory feedback.

While this design was appropriate for feasibility testing of the prototype system, the results cannot be directly generalized to clinical rehabilitation populations. Individuals with gait impairments, such as stroke survivors or those with Parkinson’s Disease, may experience different usability challenges, levels of comfort or responsiveness to auditory feedback, particularly given their need for gait adjustment and rehabilitation support. Future studies should, therefore, evaluate the system in clinical cohorts to determine its effectiveness and applicability in rehabilitation contexts. Examining the long-term effects of auditory feedback in clinical settings will be crucial for understanding its sustained impact and refining protocols for optimal rehabilitation outcomes. Additionally, integrating adaptive algorithms that align sound timing and intensity with each individual’s gait may enhance both usability and effectiveness.

## 5. Conclusions

This feasibility study demonstrated the potential of wearable auditory feedback, delivered via discrete bass and continuous whoosh sounds, to enhance movement awareness and motivation during gait activities. Rhythm-based cues such as bass tones were effective in drawing attention to specific gait phases (e.g., heel strike and initial contact), supporting coordination and motor synchronization, while continuous sounds expanded awareness to the trunk and hips, albeit with some reports of disorientation. These findings highlighted the role of auditory modality in shaping user perception and engagement with movement, demonstrating the importance of tailoring feedback to individual needs.

Quantitative analyses revealed no statistically significant differences in rotation angle between auditory feedback and walking speed conditions; however, the effect size indicated small to moderate trends in some cases, suggesting that auditory feedback may influence gait dynamics. Correlation analyses further revealed weak to moderate associations between movement parameters and participants’ self-reported motivation and awareness, indicating a measurable link between auditory cues and both biomechanical and perceptual outcomes.

The participants’ reported perceptions reinforced these observations, with 40% of participants reporting a sense of imbalance with specific sounds, while many others highlighted increased motivation and increased awareness of movement. Thematic analysis of reflections highlighted participants’ preference for greater customizability, comfort and tailored design, reinforcing the necessity for user-centered development. Although haptic feedback was not investigated in this study, it remains an important modality that can be integrated with sonification in future designs. The combination of haptic and auditory cues is likely to represent a promising trend in modern wearable devices, offering richer and more adaptive feedback options. Within this context, our findings suggest encouraging directions for auditory-based solutions, particularly when personalized and aligned with users’ perceptual and motivational needs.

By integrating real-time biomechanical monitoring with personalized auditory cues, the system demonstrated its potential to serve as a feedback tool and a source of meaningful gait parameters, which could function as digital biomarkers in future clinical applications.

The use of healthy participants and primarily subjective measures may have restricted the extent to which clinical effectiveness could be demonstrated. Nevertheless, these feasibility findings provide an evidence base for future validation in target populations, such as stroke survivors, individuals with Parkinson’s Disease, or older adults with mobility impairments. Future studies should also incorporate objective gait parameters (e.g., stride symmetry, variability, and balance measures) and long-term adherence outcomes to establish therapeutic benefit and fully assess translational potential.

## Figures and Tables

**Table 1:** Comparison of sonification vs. Rhythmic Auditory Cueing in biofeedback as an intervention to support abnormal gait and rehabilitation.

Aspect	Sonification	Rhythmic Auditory Cueing (RAC) /Rhythmic Cueing
Feedback type	Real-time, continuous; parameter-to-sound mapping [42,43]	Rhythmic, repetitive pulses or beats (isochronous cues) [25,35,44]
Information mapping	Direct/continuous mapping (e.g., pitch, loudness, timbre) to biomechanical/physiological parameters (joint angle, cadence, etc.) [45]	External tempo or beat or cue that user synchronizes movement and steps [46]
Primary mechanism	Sensorimotor integration, closed-loop adaptation, enhanced proprioception and internal feedback [47,48]	Entrainment/synchronization (phase locking to external rhythm) [44]
Adaptivity /Personalization	High: can adjust mappings, thresholds, sonification parameters per user/context [20]	Lower: usually fixed tempo/beat, limited adaptation
Use cases	Gait, joint alignment, balance, more continuous or variable tasks [20,49]	Gait, postural training, rehabilitation with rhythmic repetition [31,34]
Engagement	Supports motivational engagement, richer perceptual experience [20,39]	Strong for regular/rhythmic tasks, easier entrainment, but less flexible adaptation [44]
Strength /Limitation	Comprehensive feedback signals, supports deep sensorimotor integration, flexible [50,51]	Strong in promoting timing and regularity; less adaptive [46]

**Table 2:** Characteristics of participants (N = 20) who took part in the study.

Demographic	Mean ± Standard Deviation (SD)	Minimum	Maximum
Age (years)	35.5 ± 10.3	21	60
Height (cm)	170.9 ± 10.3	152	185
Weight (kg)	75.8 ± 14.1	52	95

## Data Availability

The data presented in this study are available on request from the corresponding author. The data are not publicly available due to privacy.

## Appendix B

**Table A1:** Shapiro-Wilk analysis to determine normality of paired tests (*t*-test was used where difference scores were approximately normal; otherwise, Wilcoxon Signed-Rank test was used) in different conditions, with the test statistic, *p*-value, effect size, and estimate with the 95% CI (mean difference for paired *t*; Hodges–Lehmann median difference for Wilcoxon).

Normality					Paired Tests						Effect Size
Walking Speed (km/h)	Pair (Difference)	Shapiro–Wilk	*p*	Interpretation	Mean Difference	Std. Deviation	95% CI of the Difference		*t* or *Z*	*p*-Value	Cohen’s d
							Lower	Upper			
2	No audio-Discrete (bass)	0.9240	0.237	Normal distribution (use paired *t* test)	−0.0055	0.0960	−0.0505	0.0394	*t* = −0.259	0.399	*d* = 0.0960
	No audio-Continuous (whoosh)	0.968	0.705	Normal distribution (use paired *t* test)	−0.0138	0.1264	−0.0729	0.0454	*t* = −0.487	0.316	*d* = 0.1264
3.9	No audio-Discrete (bass)	0.901	0.042	Not normal distribution (use Wilcoxon Signed-Rank test)	−0.0320	0.049	−0.013	0.103	Z = −1.643, W = 61	0.100	*r* = 0.1482 (Wilcoxon)
	No audio-Continuous (whoosh)	0.966	0.676	Normal distribution (use paired *t* test)	−0.0043	0.1286	−0.0645	0.0559	*t* = −0.149	0.441	*d* = 0.1286

**Table A2:** Normality checks and correlations (Pearson or Spearman with 95% CI) between mean angle of rotation and subjective ratings (motivation and awareness) under different auditory feedback and walking speeds.

Walking Speed (km/h)	Soud Condition	X (Rotation °)	Y (Subjective)	Shapiro–Wilk (Rotation)	Shapiro–Wilk (Subjective)	Decision	*r* (Effect Size)	*p*-Value	95% CI for *p*
2 km/h	Discrete (Bass)	Rotation (°)	Awareness (1–5)	0.12	0	Spearman	−0.260	0.268	[−0.674, 0.203]
	Discrete (Bass)	Rotation (°)	Motivation (1–5)	0.12	0.002	Spearman	−0.157	0.510	[−0.554, 0.314]
	Continuous (Whoosh)	Rotation (°)	Awareness (1–5)	0.101	0.007	Spearman	−0.114	0.633	[−0.541, 0.433]
	Continuous (Whoosh)	Rotation (°)	Motivation (1–5)	0.101	0.017	Spearman	0.123	0.606	[−0.352, 0.577]
3.9 km/h	Discrete (Bass)	Rotation (°)	Awareness (1–5)	0.12	0	Spearman	−0.329	0.157	[−0.759, 0.171]
	Discrete (Bass)	Rotation (°)	Motivation (1–5)	0.12	0.002	Spearman	−0.127	0.594	[−0.561, 0.309]
	Continuous (Whoosh)	Rotation (°)	Awareness (1–5)	0.162	0.007	Spearman	−0.029	0.903	[−0.499, 0.566]
	Continuous (Whoosh)	Rotation (°)	Motivation (1–5)	0.162	0.017	Spearman	0.158	0.507	[−0.329, 0.617]
